# High-Shear Mixing-Assisted Esterification of Lauric Acid to Produce Value-Added Products and Intermediaries: Effect of the Alcohol Structure

**DOI:** 10.1021/acsomega.5c04199

**Published:** 2025-09-26

**Authors:** Federico Manuel Reyes-Cruz, Manuel Sánchez-Cantú, Roberto Quintana-Solórzano, Jesús Sandoval-Ramírez, Alan Carrasco-Carballo

**Affiliations:** † Facultad De Ingeniería Química, 3972 Benemérita Universidad Autónoma De Puebla, Avenida San Claudio y 18 Sur, C.P., 72570 Puebla, México; ‡ 42592 Instituto Mexicano Del Petróleo, Eje Central Lázaro Cárdenas Norte 152, Ciudad De México 07730, México; § Laboratorio De Elucidación y Síntesis En Química Orgánica, Instituto De Ciencias, Benemérita Universidad Autónoma De Puebla, 72570 Puebla, México

## Abstract

This paper describes the benefits of incorporating the high-shear mixing (HSM) technology to intensify the homogeneous acid-catalyzed esterification of lauric acid (LA) with a set of alcohols having different chain lengths and branching degrees to produce lauric acid alkyl esters, as a sustainable alternative for producing diverse products (cosmetics, diesel fuel, cetane improvers, and chemical intermediaries). To elucidate the effect of the alcohol structure, the esterification reaction is conducted in the liquid phase at 60 °C, with an alcohol-to-LA molar ratio of 13:1 and 500 rpm for 12 min, using sulfuric acid as the catalyst. The HSM-assisted esterification requires only 12 min to suffice to convert from 88.2 to 93.6% fed LA and from 16.3 to 90.7% fed LA when reacting with linear and branched alcohols, respectively. LA conversion is generally low when using relatively simple and branched alcohols (*tert*-butanol and isopropanol) due to thermodynamic restrictions, which are ascribed to a steric hindrance effect. Notably, it is also demonstrated that the incorporation of HSM not only reduces the required time for LA conversion but also makes the reaction more efficient, thus compensating for the inherent negative dilution effect of the produced water on the liquid acid catalyst compared with the nonintensified esterification process.

## Graphical abstract

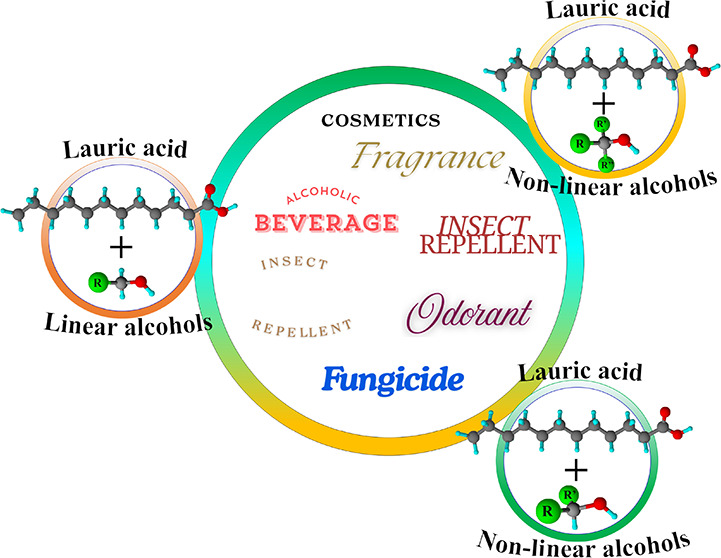

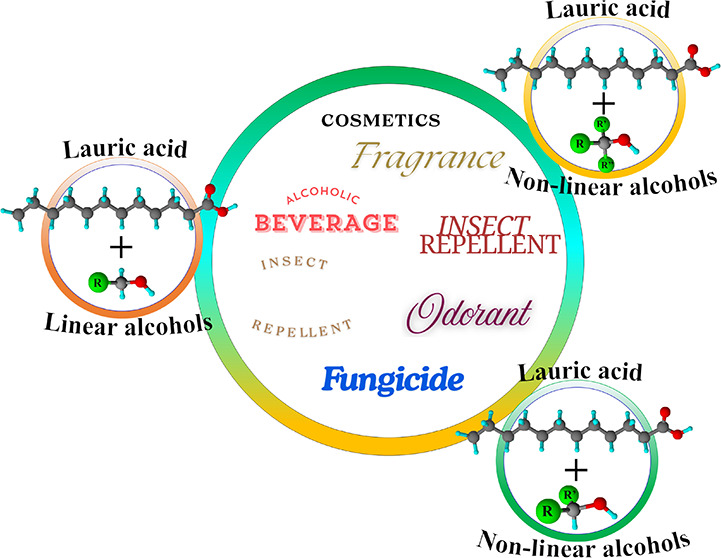

## 1 Introduction

Green chemistry (Gch) is conceived as the design of chemical processes that have the aim to eliminate or reduce the use and generation of hazardous substances,^1^ among other benefits. In this regard and bearing in mind the 12 principles of Gch, the usage of renewable feedstocks has attracted special attention in the field of energy production using biodiesel fuel, which is generally produced from vegetable oils, animal greases, and free fatty acids. Nevertheless, these feedstocks have been pigeonholed to biofuel production, restricting their potential application in other important areas. For instance, lauric acid alkyl esters (LAAEs), which are produced through the esterification of lauric acid (LA) with different types of alcohols, have attracted special attention in cosmetics,^2^ perfumery,^3^ and food flavoring^4^ industries as intermediary compounds.^5^ Some specific uses and applications of LAAEs are listed in Table S1 (see the Supporting Information file, SI file).

Contrasting with their increasing importance and demand worldwide, LAAEs are produced through processes that still experience challenges in the required reaction conditions (catalyst, reaction temperature, time, alcohol nature, or alcohol-to-fatty acid ratio). To have a complete view of this, Table S2 in the SI file displays a summary of the reported experimental conditions for LAAEs production through the esterification of AL with linear and branched alcohols. It is noted that for heterogeneous catalyzed esterification, a large excess of alcohol (alcohol/LA between 12 and 60 mol/mol), high reaction time (5–24 h), and a large catalyst amount (1.6–20 wt %) are usually needed. In the case of enzymatic or homogeneous acid-catalyzed esterification of LA, although a lower excess of alcohol is used compared with that in the heterogeneous processes, the required reaction time and catalyst amount are still large. Related to the homogeneous-phase esterification, Kastratović and Bigović studied the reaction of stearic acid with primary, secondary, and tertiary alcohols, varying temperature from 35 to 65 °C and using H_2_SO_4_ as catalyst;^6^ they found that, due to steric disturbances, the alcohol reactivity order decreased as follows: primary > secondary > tertiary alcohols. They also stated that the alcohol reactivity increased with the number of carbon atoms in the molecule, while the reaction time remained high, up to 240 min, to achieve LA conversion values of around 90%.

In the last years, intensifying reacting processes with the use of the high-shear mixing (HSM) technology has been successfully incorporated in the homogeneous transesterification of vegetable oils^7,8^ and the homogeneous esterification of free fatty acids.^9^ The benefits of HSM-assisted chemical processes are settled on the enhanced dispersion of the reactants and catalyst, which favors their intimate contact; this reduces the existing mass transfer limitations, decreases the reaction time, and increases the energy efficiency and atom economy. It was reported that by incorporating the HSM technology to intensify the homogeneous esterification reaction of LA with methanol, the reaction time declined between 5 and 120 times^9^ the required time of non-HSM-assisted processes.

In this context, although it is clear that issues related to the steric hindrance may play a key role in the reactivity of the alcohol in the esterification reaction, the combined influence of the alcohol structure and the HSM intensification on the esterification of LA needs to be formally investigated. Moreover, in recent studies, it was reported that incorporation of HSM reduces the dilution effect of the formed water in esterification reaction on the catalyst activity, thus requiring, in general, lower reaction time for a target conversion. It is therefore important to demonstrate that the use of HSM technology is suitable for the production of diverse LAAEs independently of the alcohol nature. In the framework of what was commented above, the experimental liquid phase acid-catalyzed homogeneous esterification of LA with (non)­branched alcohols with 1–5 carbon atoms is investigated in this work. With the aim of evaluating the combined effect of HSM with alcohol structure in the esterification of LA with the selected alcohols, the reaction is conducted at the same process conditions: 60 °C, alcohol/LA molar ratio of 13:1, 4.0 wt % catalyst mass, 500 rpm, and 12 min of reaction. This allows for a quantitative assessment of the advantages of assisting a set of homogeneous LA esterification processes with HSM in terms of reduced reaction times. An additional analytical novel aspect of this work lies in the identification of the esterification reaction products by ^1^H and ^13^C NMR spectroscopy which, to our knowledge, has not been reported before by this technique.

## 2 Experimental Section

### 2.1 Chemicals

Methyl alcohol (95%, Meyer), ethyl alcohol (99.5%, Meyer), propyl alcohol (99.5%, Merck), isopropyl alcohol (99.9%, Baker), butyl alcohol (99.4%, Meyer), *sec*-butyl alcohol (99.5%, Merck), isobutyl alcohol (99.4%, Chemical Products Monterrey), *tert*-butyl alcohol (99%, Baker), pentyl alcohol (98%, Chemicals Rique), and isopentyl alcohol (96.8%, Baker) were used as received. In the titration stage, potassium hydroxide (85%, pellets, Golden Bell) and phenolphthalein solution (1%, Meyer) were used. LA was acquired from Merck (≥99% purity) and heated to 120 °C for 30 min to eliminate moisture. Sulfuric acid (95%, Meyer) and deuterated chloroform (99.8 atom % D, Sigma-Aldrich) were used as catalysts and for the proton nuclear magnetic resonance (^1^H NMR) analyses. Deionized water (ca. 25 MΩ·cm) was supplied by a water deionizing plant located at the facilities of the Chemical Engineering Faculty at BUAP University.

### 2.2 Materials Characterization

#### 2.2.1 Proton Nuclear Magnetic Resonance Analysis to Confirm the Formation of Lauric Acid Alkyl Esters

The formation of the different LAAEs resulting from the esterification reactions of LA with various alcohols was verified by proton nuclear magnetic resonance (^1^H NMR) and carbon nuclear magnetic resonance (^13^C NMR). For this end, LAAEs were first mixed with deuterated chloroform (CDCl_3_) and then analyzed in a Bruker Avance III 500 MHz NMR spectrometer. The corresponding chemical shifts (δ) were expressed in parts per million (ppm).

#### 2.2.2 Acid Value (Titration) to Quantify Lauric Acid Conversion

LA conversion was indirectly calculated by means of the so-called acid value (AV) in accordance with eq 1, wherein the (*C*_LA_) is the percent LA converted in the esterification reaction, (AV_i_) corresponds to the AV value before the reaction, and (AV_f_) denotes the AV after the reaction.^10^ Similarly, the AV was determined by applying eq 2 in agreement with the method reported by Zhang et al.;^11^ *K*_A_ refers to the milliliters of potassium hydroxide solution added in the titration, *K*_B_ is the solution wasted for blank titration, *K*_m_ is the potassium hydroxide solution expressed in mol/L, *K* denotes the molecular mass of potassium hydroxide, and *M* stands for the mass taken for titration.

1 $$C_{\mathrm{LA}}\%=\frac{{\mathrm{AV}}_{i}-{\mathrm{AV}}_{f}}{{\mathrm{AV}}_{i}}\times 100$$

2 $$\mathrm{AV}=\frac{\left(K_{A}-K_{B}\right)\times -K_{m}\times K}{M}\left[=\right]\frac{\mathrm{mg KOH}}{g}$$

### 2.3 Lauric Acid Esterification Reaction

The experimental liquid phase esterification of LA with the different alcohols was carried out in batch (the corresponding reaction stoichiometry is included in the reaction schemes in Tables S3 and S4 in the SI file) at the same reaction conditions: 60 °C, 4.0 wt % catalyst mass (related to the amount of LA), 500 rpm, and 12 min of reaction time using an excess of alcohol to give an alcohol/LA molar ratio of 13:1. These process conditions were defined based on previous experimental work^9^ and considering the result of the thermodynamic analysis of the corresponding liquid phase reactions done in Aspen Plus V8 software (additional details are given in Figures S1 and S2 in the SI file in Section 4), particularly, the convenience of feeding an excess of alcohol related to which is required by stoichiometry.

Succinctly, the reaction procedure comprised the coming steps: (i) heat up the LA to 45 °C^12^ and then add the specified amount of alcohol, (ii) heat up the mixture LA–alcohol to 60 °C and then add the required quantity of sulfuric acid to catalyze the reaction, (iii) stir the reaction mixture at 500 rpm using a ROSS HSM-100 LCI high-shear mixer for the given reaction time. The apparatus consists of a single rotor equipped with a slotted stator head dispersion attachment, which was used in all experiments. As the rotating blades (fine rotor) turn around the stator, both mechanically shear the crude mixture. While in operation, mechanical shear forces producing nanodroplets favor intimate contact between the catalyst and reactants, thereby increasing the volumetric mass transfer.

Upon reaction, the unreacted alcohol was removed from the reaction mixture under reduced pressure, while the remaining mixture was washed with hot water to produce an aqueous phase containing sulfuric acid used as the catalyst. Then, the blend containing the unconverted LA, the produced alkyl ester, and water was transferred to a separation funnel to remove the aqueous fraction. The water-free mixture was next titrated (vide Section S3 in the SI file) with a potassium hydroxide solution to determine the AV according to eq 2. At the end, the LA conversion percentage was computed by eq 1. Some additional details concerning the reaction protocol and product separation are offered in the SI file in Section S3.

From a thermodynamic perspective, it is reported in the literature^13,14^ that the esterification of organic acids with alcohols is an equilibrium-limited reaction. Besides, the effect of the alcohol structure may play a key role in the esterification rate owing to the competition with the elimination reaction of organic acids with branched alcohols; in fact, when bulky alcohols are reacted, there is a steric hindrance that slows the rate of the desired esterification due to a decrease in the nucleophilic nature of the said voluminous alcohol. The thermodynamic results reported in the SI file, Section 4, confirmed that, in the esterification reaction of LA with the different alcohols, there is an equilibrium between the forward and reverse reactions. Irrespective of alcohol nature, adding an excess of alcohol favors the rate of the forward reaction, thus reducing the existing thermodynamic restrictions for LA conversion. It was also observed that the positive effect of the excess of alcohol toward the production of the corresponding ester depends upon the structure of the alcohol (carbon number and branching degree). In general, the simpler the structure of the alcohol, the more evident the increase in the yield of the ester upon addition of an excess of alcohol. Conversely, temperature has a weak effect on LA conversion values at the equilibrium independently of the alcohol nature, which is understandable considering the low values of the standard enthalpy of reaction and standard Gibbs free energy of reaction (vide SI file, Section S4).

## 3 Results and Discussion

### 3.1 Proton Nuclear Magnetic Resonance (^1^H NMR)

The formation of LAAEs from the esterification reactions using different alcohols was verified by ^1^H NMR spectroscopy after treatment to eliminate the remanent alcohol and sulfuric acid. Specifically, the signals of the alkyl esters produced in the HSM-assisted homogeneous sulfuric acid-catalyzed esterification of LA with linear and branched alcohols are displayed in Figures 1 and 2, respectively. Notice that the signals of LA have been included as a reference (the acidic proton of OH appears as a broad signal toward 10.5 ppm). In general, the evidence of the formation of LAAEs was associated with the slight displacement to lower frequencies of the triplet signal located between 2.3 and 2.4 ppm protons at C-2 of LA, and the appearance of new signals corresponding to the produced LAAEs. For instance, the formation of methyl laurate was confirmed by the singlet located at 3.54 ppm corresponding to the methyl group of –OMe. In this sense, the chemical shifts of protons of LAAEs, δ (ppm), are included in Tables 1 and 2, while the ^13^C NMR spectra and the analysis of the chemical shift of carbon can be visualized in the SI file, in Section S5; notice that the ^13^C NMR spectra are at one with the ^1^H NMR ones. The obtained ^1^H NMR spectra for LA,^15^ methyl laurate,^16^ ethyl laurate,^17^ and propyl laurate^18^ are in good agreement with information included in other reports. Likewise, it is worth commenting that, to our knowledge, the assignation signals for butyl laurate, isobutyl laurate, pentyl laurate, isopropyl laurate, *sec*-butyl laurate, *tert*-butyl laurate, and isopentyl laurate have not been reported in the literature yet.

**Figure 1:**
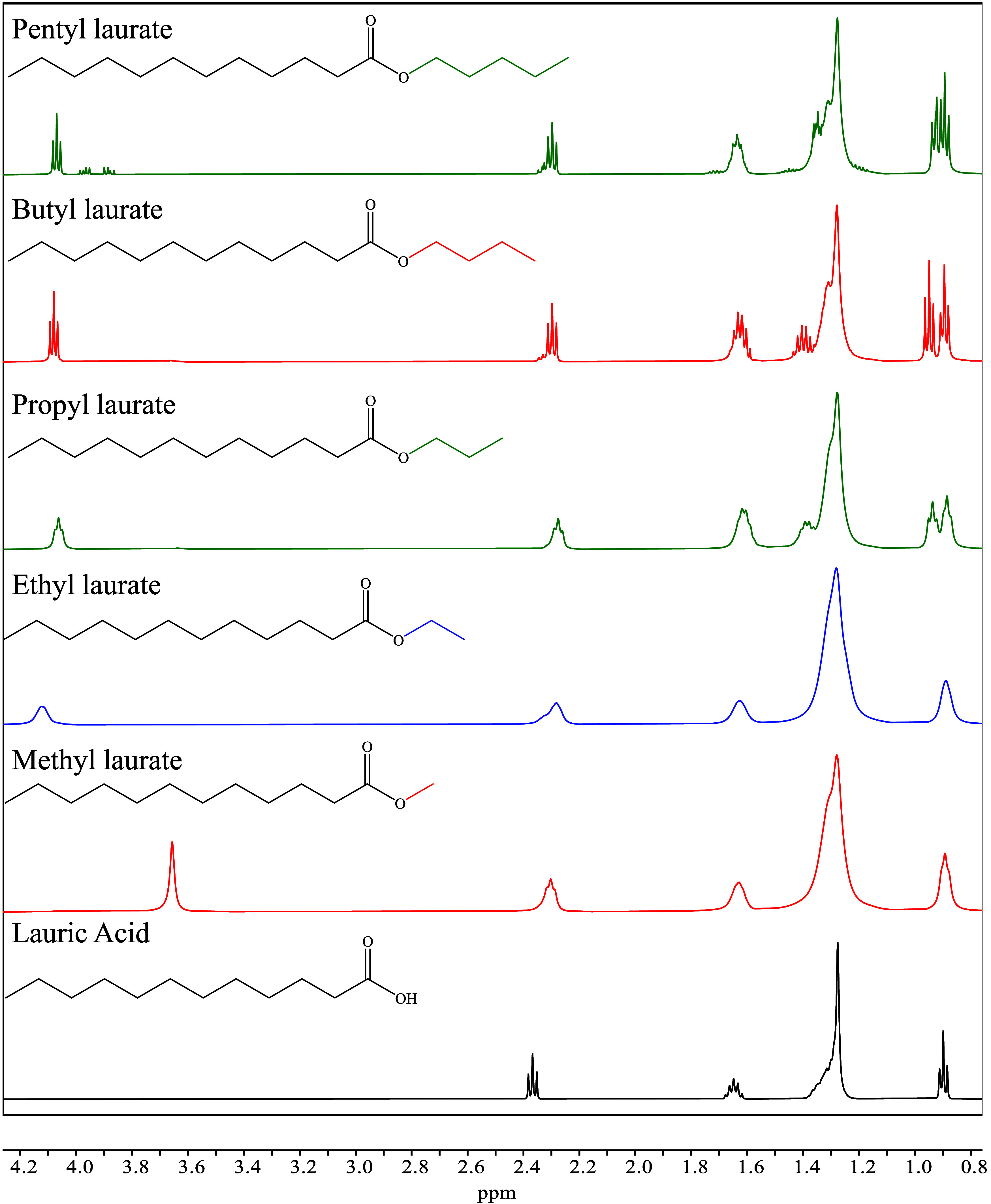
^1^H NMR spectra of the LAAEs produced from the HSM-assisted homogeneous sulfuric acid-catalyzed esterification of LA with linear alcohols.

**Figure 2:**
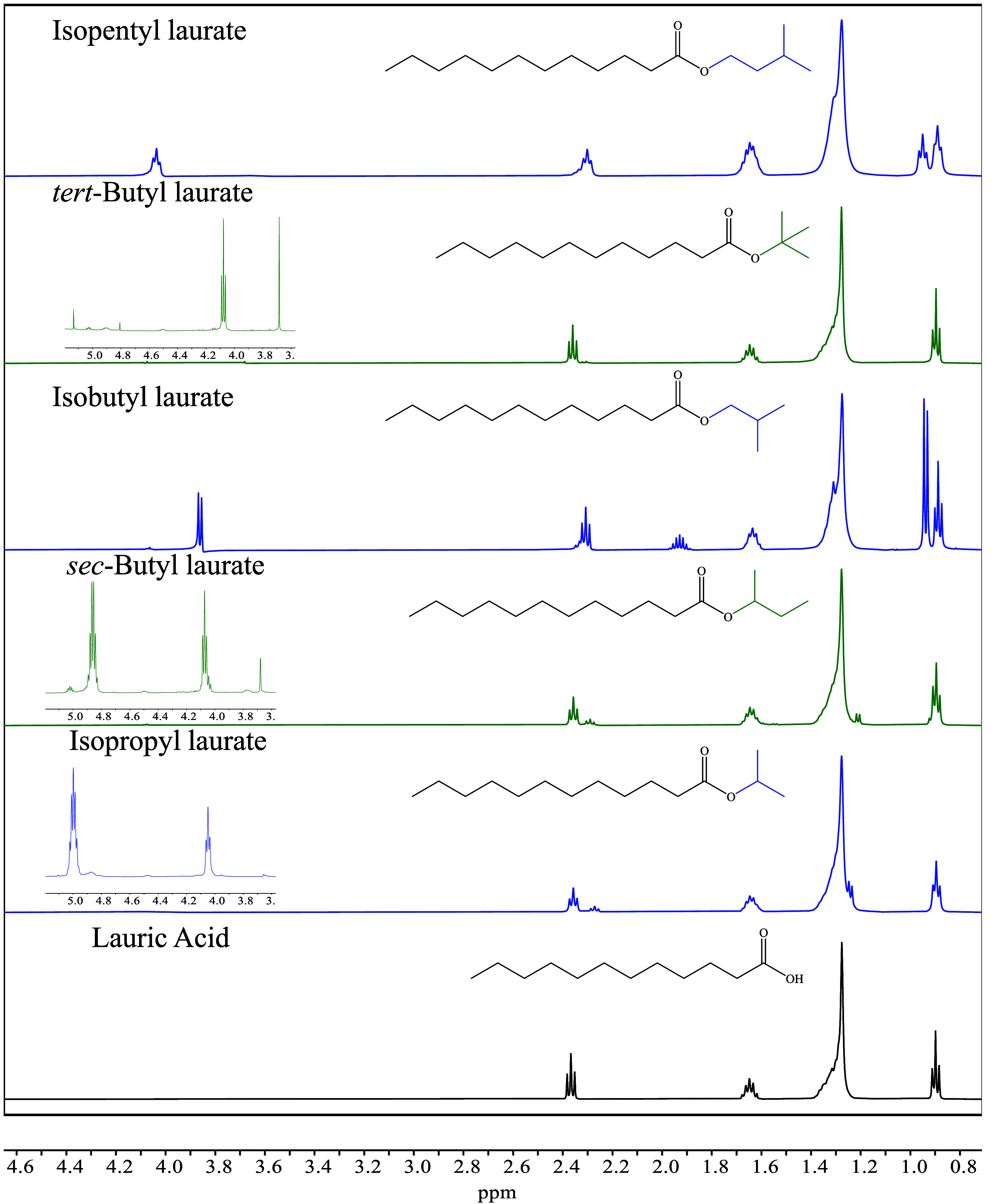
^1^H NMR spectra of the LAAEs produced from the HSM-assisted homogeneous sulfuric acid-catalyzed esterification of LA with branched alcohols.

**Table 1:** Characteristic ^1^H NMR Chemical Shifts Observed in the LAAEs Produced from the HSM-Assisted Homogeneous Sulfuric Acid-Catalyzed Esterification of LA with Linear Alcohols

	compound	chemical shifts, δ (ppm)
raw material	lauric acid	2.36 (t, *J* = 7.54 Hz, 2H, C-2), 1.64 (q, *J* = 6.33 Hz, 2H, C-3), 1.29 (m, 16H, C-4 to C-11), 0.89 (t, *J* = 6.90 Hz, 3H, C-12)
linear alcohol	methyl laurate	3.54 (s, 3H, OMe), 2.18 (t, *J* = 7.86 Hz, 2H, C-2), 1.18 (m, 16H, C-4 to C-11), 0.77 (t, *J* = 7.06 Hz, 3H, C-12)
	ethyl laurate	4.01 (t, *J* = 6.87 Hz, 2H, C-1′), 2.18 (t, *J* = 7.8 Hz, 2H, C-2 -), 1.52 (m, 2H, C-3), 1.17 (m, 16H, C-4 to C-11), 0.78 (t, *J* = 7.0 Hz, 3H, C-12)
	propyl laurate	3.92 (t, *J* = 6.77 Hz, 2H, C-1′), 2.13 (t, *J* = 7.70 Hz, 2H, C-2), 1.46 (qt, *J* = 7.10 Hz, 2H, C-2′), 1.25 (m, 2H, C-3), 1.15 (m, 14H, C-4 to C-11), 0.79 (t, *J* = 7.59 Hz, 3H, C-3′), 0.74 (t, *J* = 6.96 Hz, 3H, C-12)
	butyl laurate	4.00 (t, *J* = 6.87 Hz, 2H, C-1′), 2.18 (t, *J* = 7.55 Hz, 2H, C-2), 1.60 (m, 4H, C-3 and C-2′), 1.53 (m, 2H, C-3), 1.42(q, *J* = 6.92 Hz, CH2–3′). 1.19 (m, CH_2_–4–11), 0.83 (dd, *J* = 7.16 Hz, *J* = 3.11 Hz, CH_3_-4′), 0.79 (t, *J* = 6.95 Hz, CH_3_-12)
	pentyl laurate	3.98 (t, *J* = 6.8 Hz, CH_2_–1′), 2.22 (m, CH_2_–2), 1.55 (m, CH_2_–3,2′), 1.19 (m, CH_2_–4–11, 3′-4′), 0.82 (m, CH_3_-12)
singlet (s), doublet (d), triplet (t), quadruplet (qd), quintuplet (qt), multiplet (m)		

**Table 2:** Characteristic ^1^H NMR Chemical Shifts Observed in the LAAEs Produced from the HSM-Assisted Homogeneous Sulfuric Acid-Catalyzed Esterification of LA with Branched Alcohols

	compound	chemical shifts, δ (ppm)
branched alcohol	isopropyl laurate	5.31 (m, CH-1′), 5.05 – 4.95 (m, 1H), 4.07 (t, *J* = 6.7 Hz, 1H), 2.36 (t, *J* = 7.6 Hz, CH_2_–2), 2.28 (q, *J* = 8.4 Hz, 3H), 1.64 (m, CH_2_–3), 1.27 (m, CH_2_–4–11), 1.24 (d, *J* = 6.36 Hz, CH_3_-2′,3′), 0.89 (t, *J* = 6.8 Hz, CH_3_-12)
	*sec*-butyl laurate	4.86 (q, *J* = 6.3 Hz, CH-1′), 2.36 (t, *J* = 7.5 Hz, CH_2_–2), 1.63 (m, *J* = 7.2 Hz, CH_2_–3), 1.28 (m, *J* = 6.7 Hz, CH_2_–4–11), 1.21 (d, *J* = 6.3 Hz, CH_2_–2′), 0.90 (m, *J* = 7.3 Hz, CH_3_-12, CH_3_-3′)
	isobutyl laurate	3.80 (d, *J* = 6.64 Hz, CH_2_–1′), 2.24 (t, *J* = 7.51 Hz, CH_2_–2), 1.87 (dq, *J* = 13.39 Hz, *J* = 6.09 Hz, CH2–2′), 1.57(dd, *J* = 10.02 Hz, *J* = 4.61 Hz, CH_2_–3), 1.24 (m, CH_2_–4–11), 0.88 (d, *J* = 6.94 Hz, CH_3_-3′), 0.83 (t, *J* = 6.92 Hz, CH_3_-12)
	*tert*-butyl laurate	2.32 (t, *J* = 7.39 Hz, CH_2_–2), 1.65 (m, *J* = 7.4 Hz, CH_2_–3 and CH_3_, *tert*-butyl), 1.49 (m, CH_3_-2′-4′) 1.28 (m, CH_2_–4–11), 0.90 (t, *J* = 6.9 Hz, CH_3_-12)
	isopentyl laurate	3.98 (d, *J* = 6.76 Hz, CH_2_–1′), 2.26 (t, *J* = 7.56 Hz, CH_2_–2), 1.59 (m, CH_2_–2′, CH_2_–4), 1.24 (m, CH_2_–4–11), 0.90 (t, *J* = 7.49 Hz, CH_3_-5′), 0.84 (t, *J* = 6.88 Hz, CH_3_-12)
singlet (s), doublet (d), triplet (t), quadruplet (qd), quintuplet (qt), multiplet (m)		

### 3.2 Influence of the Alcohol Structure on the Esterification of Lauric Acid

Figure 3 displays a summary of the LA conversion values obtained from the HSM-assisted homogeneous sulfuric acid-catalyzed esterification of LA with different alcohols. The reaction was performed at 60 °C, 4.0 wt % catalyst mass, 500 rpm, 12 min of reaction time and an alcohol-to-LA molar ratio of 13.0. In general, carrying out the esterification reaction with linear alcohols leads to relatively large value of LA conversion (confidence interval limits estimated at the 95% probability are also included), viz., 90.9 (±3.4) % with methyl alcohol, 88.2 (±4.7) % with ethyl alcohol, 90.7 (±5.0) % with propyl alcohol, 92.1 (±5.7) % with butyl alcohol, and 93.6 (±4.9) % with pentyl alcohol. Regarding the LA esterification with branched alcohols, LA conversion evolved as follows: 51.5 (±5.6) % with isopropyl alcohol, 80.0 (±6.0) % with *sec*-butyl alcohol, and 76.2 (±2.3) % with isobutyl alcohol. In the case of *tert*-butyl alcohol, LA conversion was notably lower than that observed with other alcohols, reaching a value as low as 16.3 (±4.8) %. Interestingly, for the LA esterification with isopentyl alcohol, the value of LA conversion was relatively large, reaching 90.7 (±4.3) %, a value that is comparable with the values observed for the LA esterification with linear alcohols.

**Figure 3:**
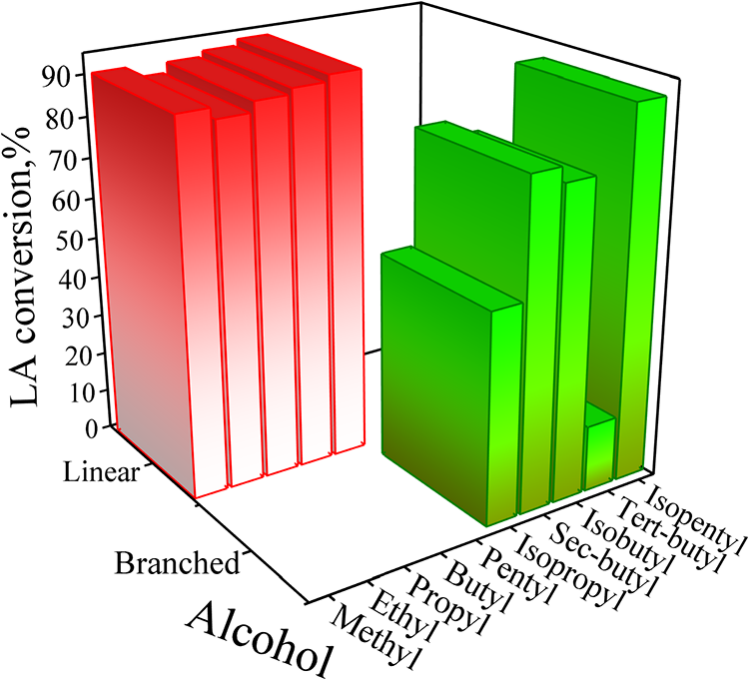
Summary of values of LA conversion in the HSM-assisted homogeneous sulfuric acid-catalyzed esterification of LA with linear and branched alcohols.

The reaction mechanism in the esterification of LA with alcohols involves five general steps that are schematically illustrated in Figure 4: (i) the acidic catalyst protons the oxygen of LA’s carbonyl, (ii) a nucleophilic attack is executed by the alcohol on the LA’s carbonyl, (iii) electron transfer occurs from oxygen atoms giving an activated complex and coproducing water, (iv) the intermediate generated accepts an alcohol’s proton and the alkyl ester is formed, and (v) secondary reactions may take place leading to the formation of alkenes.

**Figure 4:**
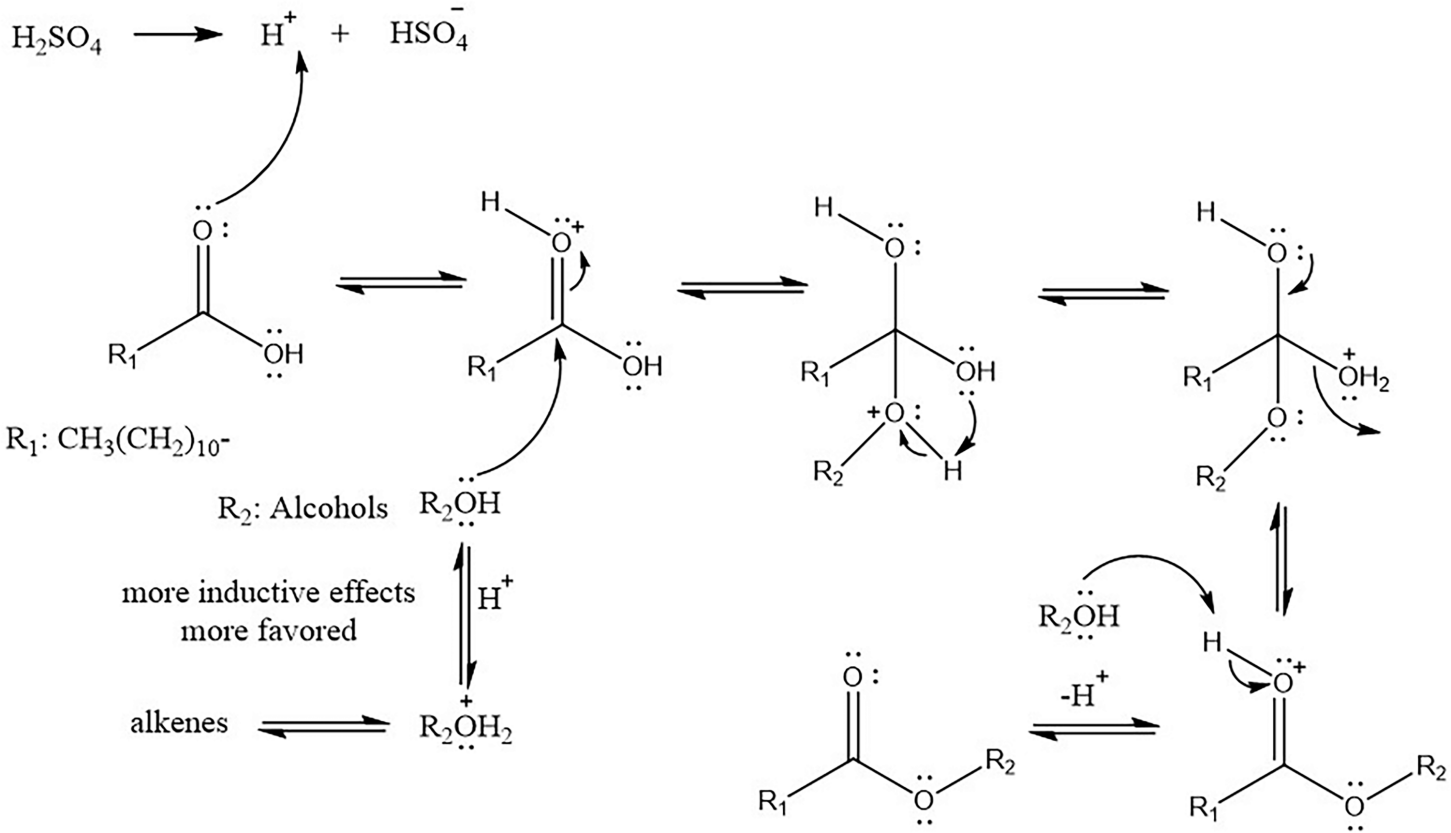
General reaction mechanism in the esterification of LA with alcohols to produce LAAEs.

Considering the series of alcohols used for the esterification of LA in this work, the reaction with branched alcohol structures, such as isopropyl and *tert*-butyl alcohol, led to the lowest values of LA conversion, a response that can be attributed to a steric hindrance effect. Nevertheless, a secondary reaction may take place during the esterification reaction leading to the formation of alkenes (vide Figure 4). The alkene formation was confirmed by the ^1^H NMR spectra in Figure 2 when performing the esterification of LA with some ramified alcohols such as isopropyl, *sec-*butyl, and *tert*-butyl. This behavior may be attributed to the use of alcohol with a larger steric impediment and, hence, its nucleophilic character decreases, which ultimately favors the rate of the elimination reaction in the acidic medium over that of the esterification.

Notice that, as reported in Table S4 in the SI file, the free Gibbs energy of reaction for the esterification of LA with *tert*-butyl alcohol has the largest positive value among the considered reactions, thereby indicating that the corresponding reaction is the most endergonic (nonspontaneous) one. Conversely, the free Gibbs energy of reaction for the esterification of LA with linear alcohols is negative (vide Table S3 in the SI file), thus implying that the forward reactions are spontaneous. In this case, the alcohol alkyl substituent incorporates easily into the LA structure by replacing the hydrogen atom in the carboxyl group to ultimately form the alkyl ester. When the alcohol structure is highly branched and the carbon chain is relatively small, as occurs in the case of isopropyl alcohol and *tert*-butyl alcohol, the alkyl substituent cannot be incorporated easily into the LA structure, and hence the formation of the corresponding ester is partially hampered. Aside, it is noted that, although isobutyl, *sec*-butyl, and isopentyl alcohols are also branched structures, the −OH group is less overshadowed by the methyl groups attached to the alcohol structure in contrast to what occurs with *tert*-butyl alcohol that is surrounded by the three methyl groups, vide Figure 5. This steric hindrance effect has been already reported in other reacting systems such as the esterification of propanoic acid,^19^ myristic acid,^20^ and oleic acid,^21^ noticing that it had a negative impact on the consumption rate of the acid and, hence, on its overall conversion.

**Figure 5:**
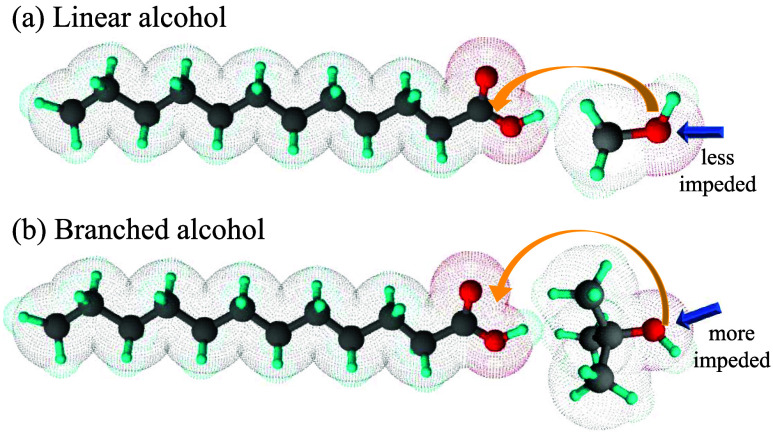
Schematic representation of the steric hindrance occurring during the acid-catalyzed homogeneous esterification of LA with alcohols with a different structure: (a) linear alcohol (methyl alcohol) and (b) branched alcohol (*tert*-butyl alcohol).

Related to the reaction severity required for the esterification of LA with the different alcohols, it is summarized in Table S2 in the SI file that, when using a conventionally mixed reactor, reaction times from 1 to 24 h are required to almost fully convert the fed LA. Interestingly, our results indicate that, at the defined set of reaction conditions (alcohol/LA molar ratio of 13:1, 60 °C, 4.0 wt % catalyst mass, 12 min, and 500 rpm), by intensifying the process with HSM, the reaction time in the homogeneous acid-catalyzed esterification of LA with the different alcohols decreased from 25 to 120 times, between 15 and 120 times, and from 8 to 20 times compared with heterogeneous, enzymatic, and homogeneous catalytic processes, respectively (see Table S7 in the SI file). As commented above, these results are ascribed to the enhanced dispersion of the reagents (LA and alcohol) and the liquid acid catalyst, which favors their intimate contact, reduces the mass transfer, and, at the end, increases the effective reaction rate.^7^

Considering the results of the thermodynamic analysis of the LA esterification with various alcohols reported in the SI file in Section S4, it was demonstrated that adding an excess of alcohol (vide Figures S1 and S2 in the SI file) helps to shift the reaction to the formation of the corresponding alkyl esters, thus reducing the thermodynamic restrictions for LA conversion. The behavior is more evident for the esterification reactions using short-chain linear alcohols such as methanol, ethanol, and n-propanol (see Figure S1). Additionally, it is important to highlight the occurrence of an additional event during the esterification reactions in our experiments. As water is invariably produced during the acid-catalyzed esterification reactions, namely, 1 mol of water per mole of LA converted according to the stoichiometry of eq (S1) in the SI file, it can decrease the catalyst efficiency by a dilution effect due to the affinity of H_2_SO_4_ and H_2_O.^9^ Although in the present study the water effect on the catalyst was not systematically evaluated for LA esterification with the various alcohols, in our previous work, it was demonstrated that the HSM attenuates the catalyst dilution by the water produced in reaction when performed in batch systems.^9^ Hence, it seems that by incorporating the HSM, the negative effect of water was somehow reduced, and relatively large values of LA conversion were achieved even when operating at relatively low reaction times.

## 4 Conclusions

The high-shear mixing (HSM), which is a promising intensified technology that represents an option to the conventional reacting process, was used to efficiently assist the liquid phase esterification of lauric acid (LA) with alcohols with different carbon numbers and branching degrees. In agreement with the governing thermodynamics, it was experimentally proven that the addition of an excess of alcohol was required to favor the production of the corresponding alkyl ester, a response that was even more evident in the case of relatively complex branched alcohols. Although the esterification of fatty acids is a well-established process, assisting the reaction with HSM provided a very efficient contact between the reagents and catalyst, thereby requiring a moderate mixing rate and time without detriment in LA conversion, i.e., 88.2–93% when using linear alcohols and 16.3–90.7% when reacting branched alcohols. In batch reacting systems, time is a determining factor for LA conversion; hence, by applying the HSM technology in this work, comparatively, reaction times were reduced between 7.5 and 120 times for linear alcohols and from 15 to 120 times for branched alcohols. It was also noticed that the inevitable diluting effect of formed water on the LA consumption rate was partially attenuated by applying HSM, which induces a very intimate contact between the reagents and catalyst. Hereafter, the use of HSM represents a promising tool to intensify the esterification reaction between LA and alcohols, which has the potential to be scaled up to produce value-added esters, offers substantial reductions in reaction time, eliminates the need for costly equipment, and provides valuable insights into how molecular structure governs reaction performance.

## Supplementary Material



## Notes

Experimental procedure details, chemical equilibrium calculations and results, and ^13^C NMR characterization and comparative results of this work with literature reports (PDF)

## Nomenclature

^1^H NMR: proton nuclear magnetic resonance
^13^H NMR: carbon nuclear magnetic resonance
AE: alkyl ester
AV: acid value (mg KOH) g^–1^
AV_i_: acid value before reaction (mg KOH) g^–1^
AV_f_: acid value after reaction (mg KOH) g^–1^
C_LA_: LA converted during the esterification reaction (%)
HSM: high-shear mixing
*K*: molecular mass of potassium hydroxide
*K*_A_: potassium hydroxide solution (ml)
*K*_B_: solution required for blank solvent titration (ml)
*K*_m_: molarity of the potassium hydroxide solution (mol/L)
LA: lauric acid
LAAEs: lauric acid alkyl esters
Alcohol/LA: alcohol/lauric acid molar ratio (mol/mol)
*M*: mass of titration (g)
rpm: revolution per minute
SI: supporting information
