# Supplementary material for: High-Shear Mixing-Assisted Esterification of Lauric Acid to Produce Value-Added Products and Intermediaries: Effect of the Alcohol Structure
Source: ACS Omega. 2025 Sep 26;10(39):45144–51. doi: 10.1021/acsomega.5c04199 (PMC12508932; doi:10.1021/acsomega.5c04199)
Supplement: Supplementary file 1 [file ao5c04199_si_001.pdf]

Supporting Information

High-Shear Mixing Assisted Esterification of Lauric Acid to Produce Value-Added Products and Intermediaries: Effect of Alcohol Structure

Federico Manuel Reyes-Cruz[(a)], Manuel Sánchez-Cantú[(a)], Roberto Quintana-Solórzano[(b)]*, Jesús Sandoval-Ramírez[(c)], Alan Carrasco-Carballo[(c)]*

[(a)] Facultad de Ingeniería Química, Benemérita Universidad Autónoma de Puebla, Facultad de Ingeniería Química, Avenida San Claudio y 18 Sur, C.P. 72570 Puebla, Puebla, México

[(b)] Instituto Mexicano del Petróleo, Eje Central Lázaro Cárdenas Norte 152, Ciudad de México, 07730, México

[(c)] Laboratorio de Elucidación y Síntesis en Química Orgánica, Instituto de Ciencias, Benemérita Universidad Autónoma de Puebla, 72570 Puebla, México

** To whom correspondence should be addressed:*

+ (52 55) 2295500 Ext. 7265; e-mail: manuel.sanchez@correo.buap.mx,

+ (52 55) 9175 8530; e-mail: rquintana@imp.mx

Key Words: intensified processes, high-shear mixing, lauric acid esterification, effect of alcohol, alcohol structure

Section S1. List of lauric acid alkyl esters and their use in different industrial activities.

In Table S1 the applications of the various lauric acid alkyl esters (LAAE) reported in the literature are listed.

Table S1. Reported applications of LAAE in diverse industrial activities

LAAEs	Constituents, and applications
Methyl laurate	High-cetane biofuel [1], insecticide, fungicide, nematicide and/or miticide [2,3], animal feed additive [4], insect repellent [5], cosmetics [6]
Ethyl laurate	High-cetane biofuel [1], tuberose essence with fruity odour [7], flavor or fragrance in food and cosmetic industries [8], volatile compounds in alcoholic beverages [9–12], skin and household care products [13,14], animal feed additive [4], coffee distilled beverage[15]
Propyl laurate	High-cetane biofuel [1], animal feed additive [4], volatile compounds in alcoholic beverages [11,12]
Butyl laurate	High-cetane biofuel [1], lubricant [16], animal feed additive[4], formulations against susceptible pathogens [17]
Pentyl laurate	Formulations against susceptible pathogens [17], in ointment for therapeutic use [18], odorant[19]
Isopropyl laurate	Odorant in the perfume industry [20], insecticide, fungicide, nematicide and/or miticide [2,3], volatile compounds in alcoholic beverage [12], formulations against susceptible pathogens[17]
sec-Butyl laurate	Insect sex pheromone [21], nutritional supplement [22]
Isobutyl laurate	Textile lubricant [23], insecticide, fungicide, nematicide and/or miticide [2,3], volatile compounds in alcoholic beverage [12]
tert-Butyl laurate	Potential antimicrobial activity [24], in ointment for therapeutic use [25]
Isopentyl laurate	Volatile compounds in alcoholic beverage [9,12], emollient in the cosmetic industry [26], insecticide, fungicide, nematicide and/or miticide[2,3], formulations against susceptible pathogens [17]

Section S2. Reported conditions for catalyzed LA esterification to LAAEs

In Table S2 shows the esterification reaction conditions of linear and branched alcohols to obtain different LAAEs reported in the literature.

Table S2. Reaction conditions reported in the state-of-the-art for the catalyzed esterification of LA with different alcohols to produce LAAEs

	Catalyst	Alcohol	Temperature, ˚C	Time, h	Catalyst, wt %	Alcohol/LA, mol/mol	Conversion, %
Heterogeneous	SiO_2@20PMoV[27]	Methyl	78	5	3.1	62	~ 55
	Montmorillonite [28]	Methyl	160	2	12	12	96.67
	SiO_2@20PMoV [27]	Ethyl	78	5	3.1	43	Above 95
	Nanostructured KIT-6 [29]	Propyl	100	24	1.6	10.9	Above 50
	SiO_2@20PMoV[27]	Isopropyl	78	5	3.1	32.6	~95
	Nanostructured KIT-6[29]	Butyl	120	24	1.6	10.9	~90
	SiO_2@20PMoV [27]	*sec*-Butyl	78	5	3.1	27.1	90
	SiO_2@20PMoV [27]	*tert*-Butyl	78	5	3.1	25.5	44
	SiO_2@20PMoV [27]	Isopentyl	78	5	3.1	23	Above 95
Enzymatic	*Aspergillus flavus*[30]	Ethyl	40	24	20 mg lipase/mL substrate	2	96
	Lipase *Mucor miehei* immobilized on chitosan hydrogels [31]	Propyl	30	24	0.15 mg/g of gel	1	60
	Rhizopus oryzae [26]	Isopentyl	45	24	350 units of immobilized lipase	1.5	81.86
	Novozym 435 [32]	Isopentyl	50	3	0.5	3	~37
Homogeneous	Ammonium ferric sulphate [33]	Methyl	65	1.5	8	6	99.8
	Sulfuric acid [34]	Ethyl	70	1.7	0.66	9	~ 88
	Sulfuric acid[35]	Ethyl	70	2.9	0.66	9	~ 85

Aryl imidazolium ionic liquid[36]	Propyl	80	4	2 mol	7	93
Sulfuric acid[37]	Butyl	110-120	4	5	1.2	93

Section S3. Experimental procedure

Apparatus: The stator head dispersion attachment was introduced in the batch reactor and then connected to reactor nozzle with an airtight sleeve stopper and Parafilm® to avoid alcohol vaporization.

Acidity assessment: Upon reaction, the excess of alcohol was removed by reducing pressure making the two non-miscible phases visible. The top layer was a mixture of LAAE and unreacted lauric acid (LA), and the bottom layer corresponded to an aqueous phase containing sulfuric acid aqueous phase. The bottom layer was removed, and the upper layer was washed with hot deionized to eliminate most of the sulfuric acid. Then, the acid value (AV) was determined for the washed upper layer. For this end, the standard method Cd 3d-63 validated by the American Oil Chemists' Society (AOCS) [38] and used to quantify the AV in fats and oils was used. The AV results were compared with instrumental techniques such as ^{1}H NMR obtaining similar values [39, 40].

Section S4. Chemical equilibrium calculations for various LA esterification reactions

In this work, the homogeneous acid catalyzed esterification reaction of LA with linear and branched alcohols with 1 to 5 carbon atoms was experimentally investigated. Said reaction was intensified by incorporating high share mixing with the corresponding results being presented and discussed in the main article.

The general esterification reaction for alcohols and LA is summarized by Eq. S1, where R, R', R'' = H (methanol); R', R''= H (primary alcohol); R'= H (secondary alcohol); R, R', R''= alkyl

(tertiary alcohol)[41]. According to the corresponding stoichiometry, the reaction of 1 mol of LA with 1 mol of a given alcohol produces 1 mol of LAAE and 1 mol of water.

Thermodynamics information of a selection of liquid phase esterification reactions of LA with different alcohols was obtained in Aspen Plus V8.8. The effect of temperature and alcohol/LA ratio, which are very important independent variables for the reactor operation, was investigated with some detail. From this, a set of graphics of LA equilibrium conversion were built.

Values of standard enthalpy of reaction and free Gibbs energy for the different esterification reactions of LA with linear and branched alcohols were also obtained and are summarized in Tables S3 and S4. Figures S1 and S2 display the values of LA conversion at equilibrium for linear and branched alcohols, respectively, at different temperatures and alcohol/LA ratios.

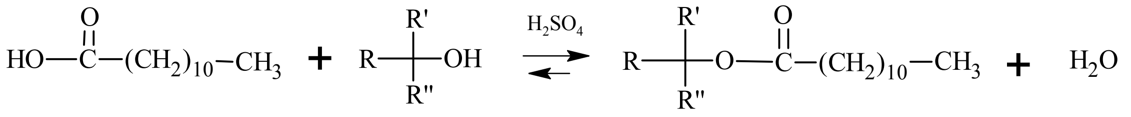

(S1)

Lauric acid **Alcohol** **Lauric Acid Alkyl Ester** **Water**

In the esterification of LA with linear alcohols (see Table S3, reactions S2-S6), it is noted that the values of the standard enthalpy of reaction are negative for methanol and ethanol, and positive for n-propanol, n-butanol and n-pentanol.

Likewise, the standard Gibbs energy values for the esterification of LA with linear alcohols are all negative; those associated to the reaction of LA with methanol and ethanol are, in fact, the most negative ones. This indicates that these chemical processes are exergonic and spontaneous and, therefore, an increase in temperature will have a negative impact on the LA equilibrium conversion.

Figure S1 shows values of LA conversion at equilibrium at varying temperatures (from 20 to 60 °C) and alcohol/LA ratio (1/1 to 13/1 mol/mol).

Table S3. Values of the standard enthalpy of reaction and standard Gibbs energy for the esterification reactions of LA with linear alcohols containing 1 to 5 carbon atoms

Alcohol	$\Delta H^{\circ}_{R,25°C}$ kJ mol^{-1}	$\Delta G^{\circ}_{R,25°C}$ kJ mol^{-1}	Reaction stoichiometry	
Methanol	-1.6	-10.9	$CH_3(CH_2)_{10}COOH + CH_3OH \rightleftharpoons CH_3(CH_2)_{10}COOCH_3 + H_2O$	(S2)
Ethanol	-2.4	-11.0	$CH_3(CH_2)_{10}COOH + C_2H_5OH \rightleftharpoons CH_3(CH_2)_{10}COOC_2H_5 + H_2O$	(S3)
n-Propanol	1.4	-4.8	$CH_3(CH_2)_{10}COOH + C_3H_7OH \rightleftharpoons CH_3(CH_2)_{10}COOC_3H_7 + H_2O$	(S4)
n-Butanol	3.4	-5.3	$CH_3(CH_2)_{10}COOH + C_4H_9OH \rightleftharpoons CH_3(CH_2)_{10}COOC_4H_9 + H_2O$	(S5)
n-Pentanol	3.8	-5.2	$CH_3(CH_2)_{10}COOH + C_5H_{11}OH \rightleftharpoons CH_3(CH_2)_{10}COOC_5H_{11} + H_2O$	(S6)

Figure S1 shows that LA equilibrium conversion is notably influenced by the LA/alcohol ratio; in general, adding an excess of alcohol favors the rate of the forward reaction producing the corresponding ester. When the number of carbon atoms in the alcohol structure is increased, a lower LA equilibrium conversion can be reached at the same condition of temperature and alcohol amount. At 60 °C, for instance, it is observed in Figure S1 that almost complete LA conversion is achieved for an alcohol/LA ratio of 4.0 when using methanol or ethanol, while a maximum LA conversion of 73 % is reached when using n-pentyl alcohol.

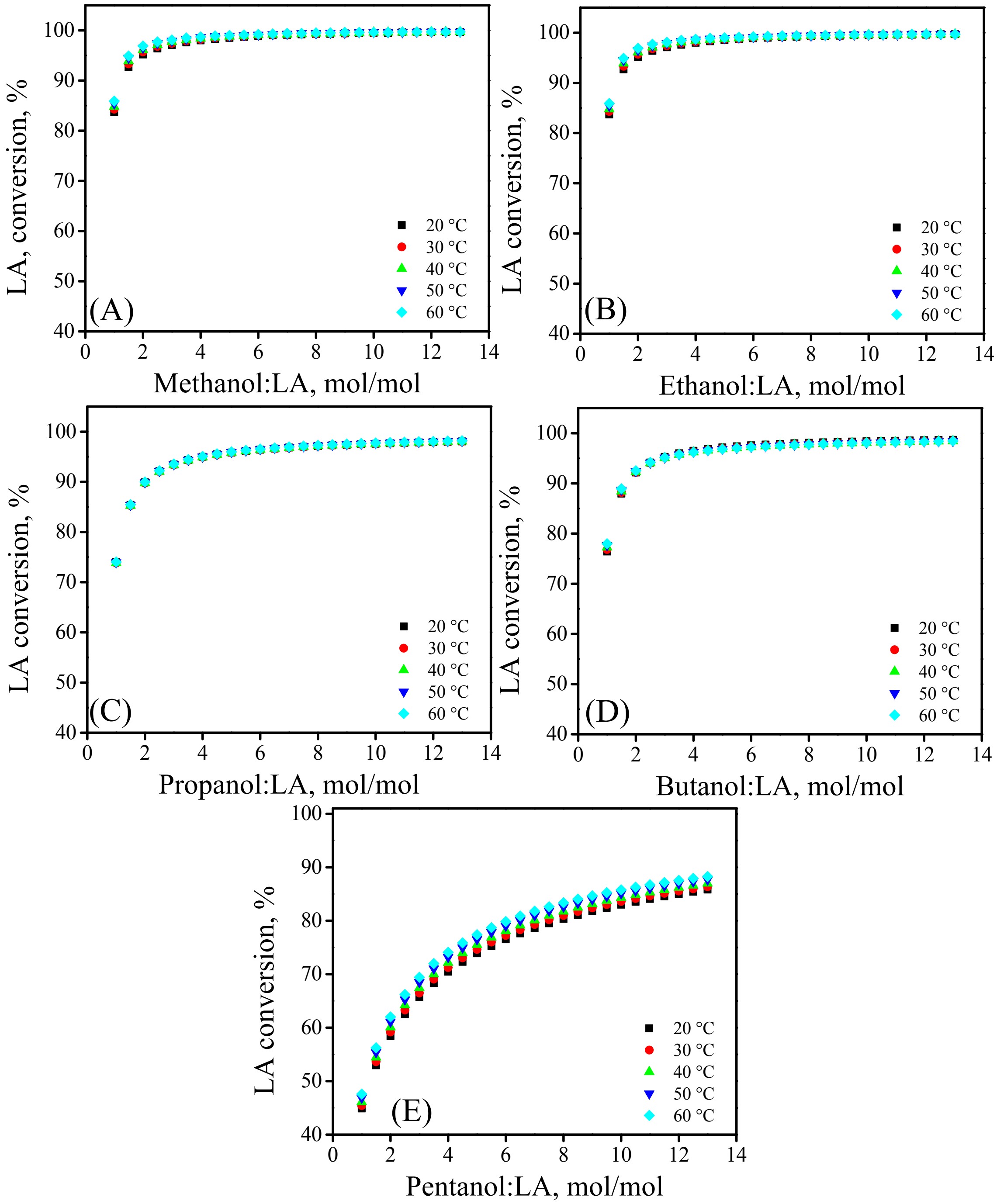

Figure S1. LA equilibrium conversion values for the esterification of LA with various linear alcohols: (A) Methanol, (B) Ethanol, (C) Propanol, (D) Butanol, and (E) Pentanol. Temperature varied from 20 to 60 °C and the alcohol/LA ratio from 1/1 to 13/1 mol/mol.

In the case of the esterification of LA with branched alcohols, the thermodynamic information displayed in Table S4 shows that values of standard enthalpy of reaction are all positive, thus indicating that these reactions are endothermic. The standard Gibbs free-energy is positive for the LA esterification with isopropanol, *sec*-butanol and *tert*-butanol (endergonic and non-spontaneous), and negative with isopentanol. In the latter case, it must be highlighted that this is a branched alcohol. For the reaction of LA with isobutanol, the primary hydroxyl group is hindered by the isopropyl branch, which would explain the observed thermodynamic restrictions for LA conversion when using branched alcohols.

Table S4. Values of the standard enthalpy of reaction and standard Gibbs energy for the esterification reactions of LA with non-linear alcohols containing 3 to 5 carbon atoms

Alcohol	$\Delta H°_{R,25°C}$ kJ mol^{-1}	$\Delta G°_{R,25°C}$ kJ mol^{-1}	Reaction	
Isopropanol	4.2	8.8	$CH_3(CH_2)_{10}COOH + C_3H_7OH \rightleftharpoons CH_3(CH_2)_{10}COOC_3H_7 + H_2O$	(S7)
sec-Butanol	4.9	8.7	$CH_3(CH_2)_{10}COOH + C_4H_9OH \rightleftharpoons CH_3(CH_2)_{10}COOC_4H_9 + H_2O$	(S8)
Isobutanol	4.9	2.3	$CH_3(CH_2)_{10}COOH + C_4H_9OH \rightleftharpoons CH_3(CH_2)_{10}COOC_4H_9 + H_2O$	(S9)
tert-Butanol	4.4	24.2	$CH_3(CH_2)_{10}COOH + C_4H_9OH \rightleftharpoons CH_3(CH_2)_{10}COOC_4H_9 + H_2O$	(S10)
Isopentanol	4.4	-3.0	$CH_3(CH_2)_{10}COOH + C_5H_{11}OH \rightleftharpoons CH_3(CH_2)_{10}COOC_5H_{11} + H_2O$	(S11)

The Fischer esterification reaction is driven to products basically by the influence of the excess of alcohol at a given temperature [42]. However, the alcohol nature has a clear effect on the thermodynamic equilibrium. It was observed that for low molecular-mass linear alcohols such as

methanol, ethanol and propanol, LA equilibrium conversion is reached when using a moderate excess of alcohol/LA ratio (4.0 to 6.0).

In the case of larger molecular-mass linear alcohols (n-pentanol, Figure S1) or branched alcohol such as isopropanol (Figure S2), a larger excess of alcohol/LA ratio (= 13) is required to overcome thermodynamic restriction for LA conversion (37 %). From an economic point of view, using a large excess of alcohol can have a negative impact on the economy of the process due to the cost of the raw material quantity and the reactor volume.

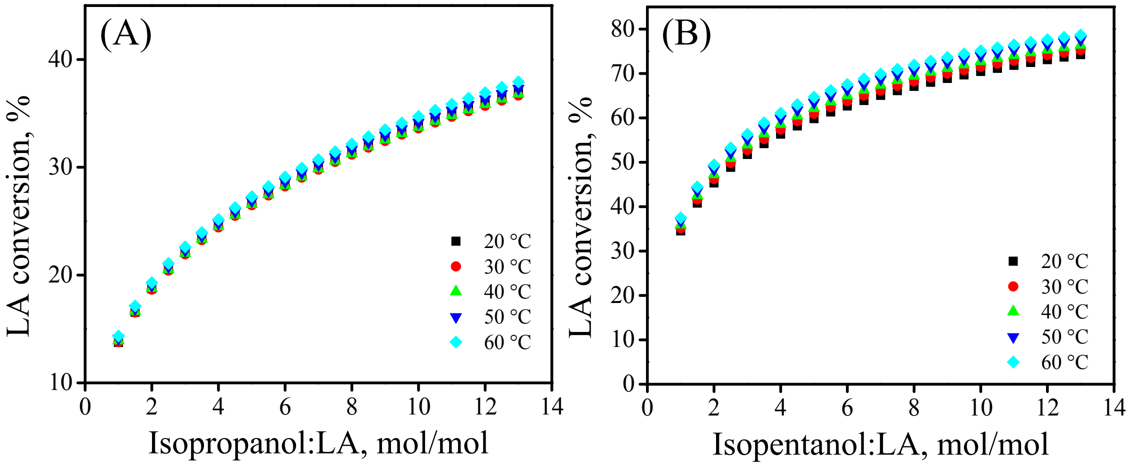

Figure S2. LA equilibrium conversion values for the esterification of LA with branched alcohols: (A) Isopropanol, and (B) isopentanol. Temperature was varied from 20 to 60 °C, and the alcohol/LA ratio from 1/1 to 13/1 mol/mol.

Section S5. Carbon Nuclear Magnetic Resonance ^{13}C NMR characterization.

The ^{13}C NMR chemical shifts of the synthesized LAAE are summarized in Tables S5 and S6 while their respective spectra for linear and branched alcohols are visualized in Figure S, respectively. The LA spectra was included at each graphic to visualize the generated changes.

Table S5. Characteristic ^{13}C NMR chemical shifts of LAAES produced from linear alcohols

	Compound	Chemical shifts, δ (ppm)
	Lauric acid	180.36 (C1), 34.11 (C2), 31.91 (C10), 29.60 (C7), 29.60 (C8), 29.43 (C4), 29.32 (C9), 29.24 (C5), 29.06 (C6), 24.67 (C3), 22.68 (C11), 14.08 (C12),
Linear alcohol	Methyl laurate	173.84 (C1), 51.01 (C1'), 33.84 (C2), 31.81(C10), 29.51 (C7), 29.51 (C7), 29.36 (C5), 29.23 (C9), 29.14 (C4), 29.04 (C6), 24.80 (C3), 22.54 (C11), 13.84 (C12)
	Ethyl laurate	173.50 (C1), 59.86 (C1'), 34.13 (C2), 31.81 (C10), 29.51 (C7), 29.51 (C8), 29.37 (C4), 29.25 (C5), 29.18 (C9), 29.04 (C6), 24.84 (C3), 22.55 (C11), 14.00 (C2'), 13.86 (C12)
	Propyl laurate	173.37 (C1), 63.71 (C1'), 34.05 (C2), 32.78 (C10), 30.60 (C7), 29.48 (C8), 29.39 (C4), 29.21(C5), 29.15 (C9), 29.01 (C6), 24.83 (C3), 22.51 (C2'), 18.98 (C11), 13.80 (C12), 13.40 (C3')
	Butyl laurate	173.52 (C1), 62.59 (C1'), 37.32 (C2), 34.17 (C10), 31.82 (C2'), 29.51 (C7), 29.51 (C8), 29.38 (C5), 29.25 (C4), 29.18 (C9), 29.06 (C6), 24.95 (C3), 22.56 (C11), 22.26 (C3'), 13.89 (C4'), 11.00 (C12)
	Pentyl laurate	173.52 (C1), 62.59 (C1'), 37.32 (C2), 34.17 (C10), 31.82 (C2'), 29.51 (C7), 29.51 (C8), 29.38 (C5), 29.25 (C4), 29.18 (C9), 29.06 (C6), 24.95 (C3), 22.56 (C11), 22.26 (C3'), 13.89 (C4'), 11.00 (C12)

Table S6. Characteristic ^{13}C NMR chemical shifts of LAAEs produced from branched alcohols

	Compound	Chemical shifts, δ (ppm)
Branched alcohol	Isopropyl laurate	180.36 (C1), 34.11 (C2), 31.91 (C10), 29.60 (C7), 29.60 (C8), 29.43 (C4), 29.32 (C9), 29.24 (C5), 29.06 (C6), 24.67 (C3), 22.68 (C11), 14.08 (C12)
	sec-Butyl laurate	180.36 (C1), 34.11 (C2), 31.91 (C10), 29.60 (C7), 29.60 (C8), 29.43 (C4), 29.32 (C9), 29.24 (C5), 29.06 (C6), 24.67 (C3), 22.68 (C11), 14.08 (C12)
	Isobutyl laurate	173.70 (C1), 70.21 (C1'), 34.22 (C2), 31.85 (C10), 29.54 (C7), 29.54(C8), 29.41 (C4), 29.27 (C5), 29.21 (C9), 29.10 (C6), 27.66 (C2'), 24.96 (C3), 22.60 (C11), 18.94 (C3'), 13.94 (C12)
	tert-butyl laurate	180.36 (C1), 34.11 (C2), 31.91 (C10), 29.60 (C7), 29.60 (C8), 29.43 (C4), 29.32 (C9), 29.24 (C5), 29.06 (C6), 24.67 (C3), 22.68 (C11), 14.08 (C12)
	Isopentyl laurate	173.80 (C1), 65.68 (C1'), 35.2 (C2'), 34.26 (C2), 31.86(C10), 29.55 (C7), 29.55 (C8), 29.41 (C4), 29.29 (C5), 29.22(C9), 29.10 (C6), 24.95 (C3), 22.61 (C3'), 21.94 (C4'), 13.96 (C11), 10.25 (C12)

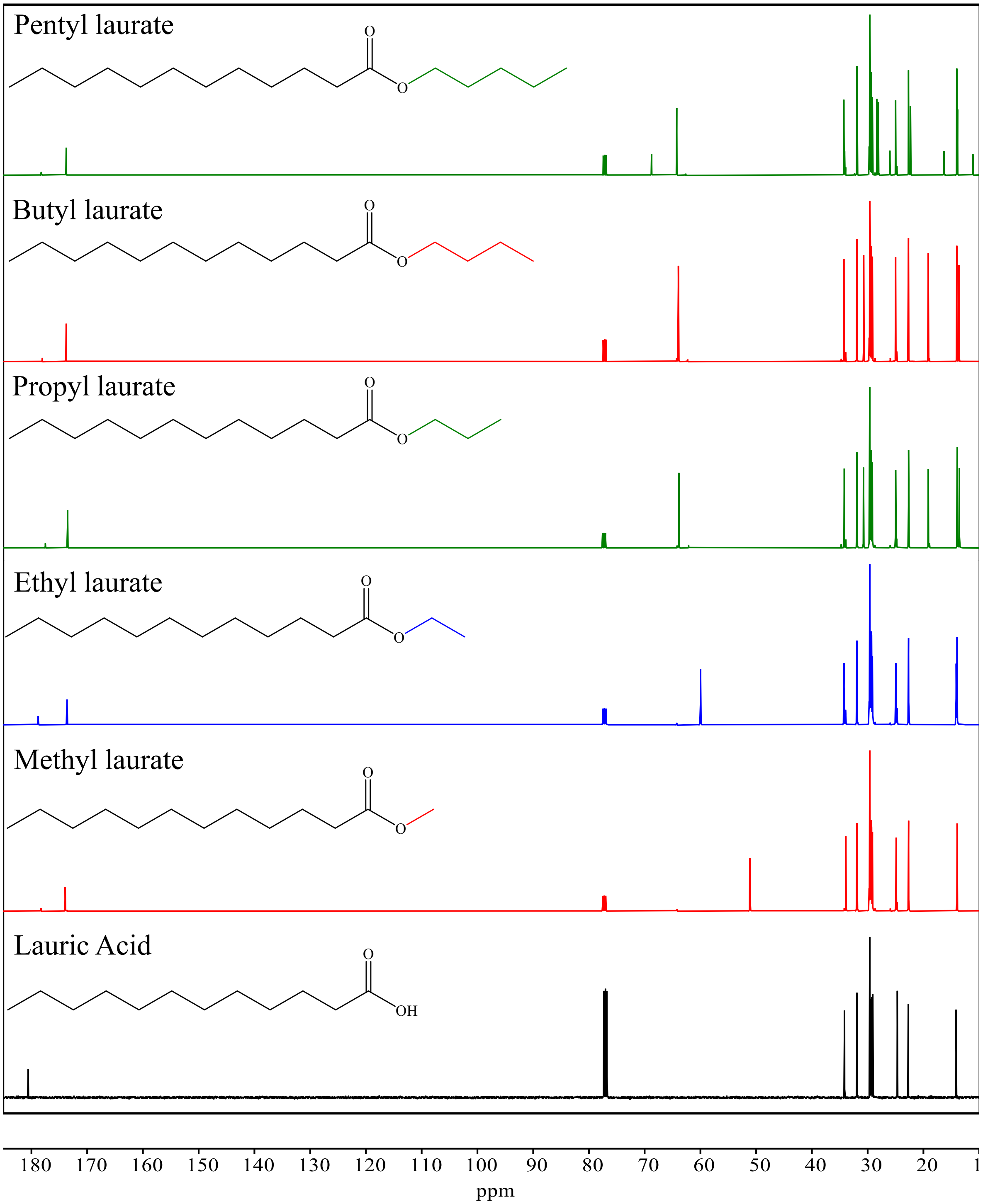

Figure S3. ^{13}C NMR spectra generated in the homogeneous esterification of LA with linear alcohols and sulfuric acid as catalyst to produce LAAE.

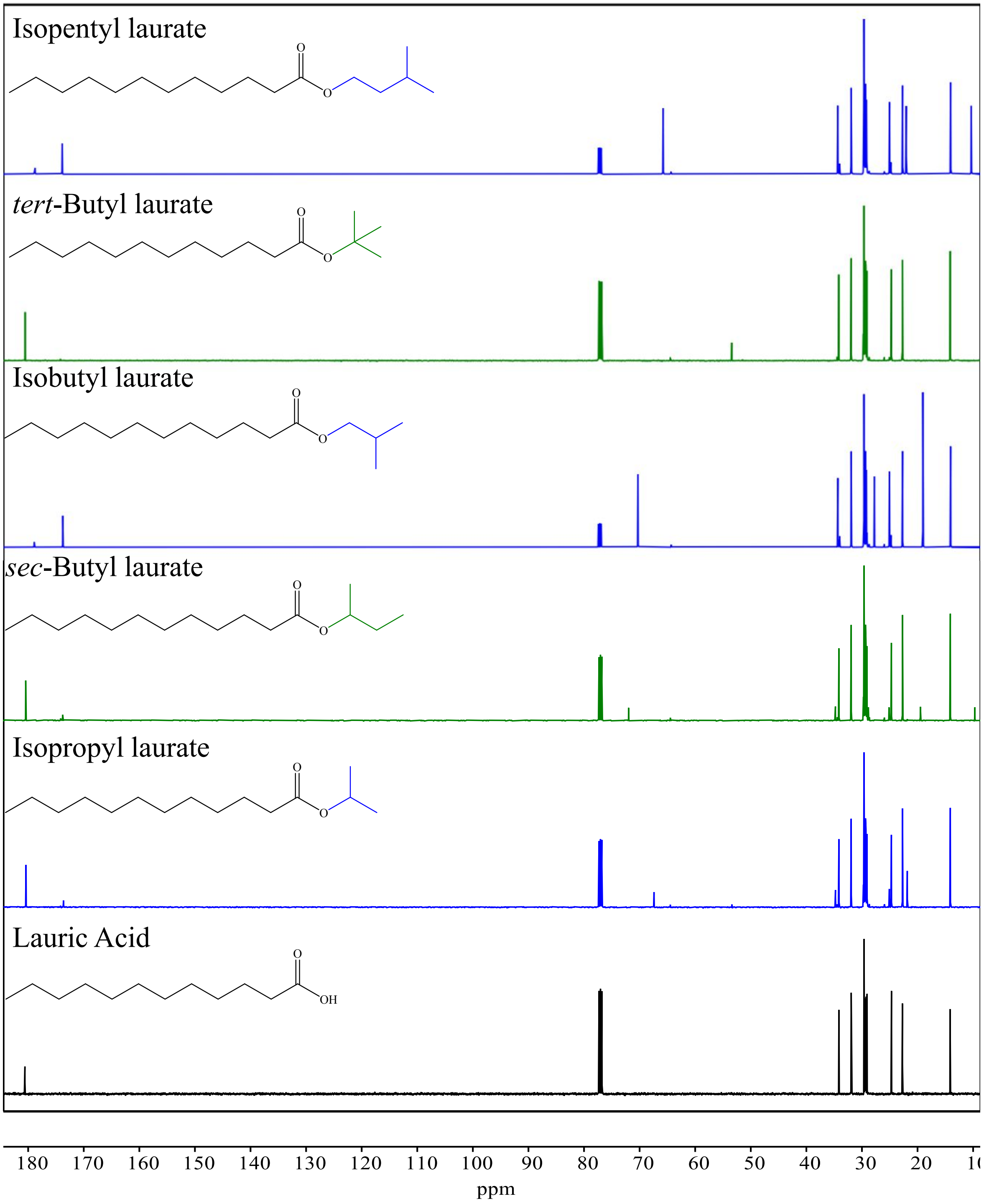

Figure S4. ^{13}C NMR spectra generated in the homogeneous acid catalyzed esterification of LA with branched alcohols to produce LAAEs.

Section S6. Comparison of reaction severity for Conventional and HSM- Intensified process in catalyzed AL esterification with diverse alcohols.

Table S7 contextualizes the significance of the present findings with respect to results reported in previous studies.

Table S7. Comparison of required reaction time and produced AL conversion during its esterification with various alcohols: Conventional vs HSM- Intensified process

			Literature		Our work (HSM assisted)	
	Catalyst	Alcohol	Time, h	Conversion, %	Time reduction, h	Conversion, %
Heterogeneous	SiO_2@20PMoV[27]	Methyl	5	~ 55	4.8	90.9
	Montmorillonite [28]	Methyl	2	96.67	1.8	90.9
	SiO_2@20PMoV [27]	Ethyl	5	Above 95	4.8	90.7
	Nanostructured KIT-6 [29]	Propyl	24	Above 50	23.8	92.1
	SiO_2@20PMoV[27]	Isopropyl	5	~95	4.8	51.5
	Nanostructured KIT-6[29]	Butyl	24	~90	23.8	93.6
	SiO_2@20PMoV [27]	*sec*-Butyl	5	90	4.8	80
	SiO_2@20PMoV [27]	*tert*-Butyl	5	44	4.8	16.3
	SiO_2@20PMoV [27]	Isopentyl	5	Above 95	4.8	90.7
Enzymatic	*Aspergillus flavus*[30]	Ethyl	24	96	23.8	90.7
	Lipase *Mucor miehei* immobilized on chitosan hydrogels [31]	Propyl	24	60	23.8	92.1
	Rhizopus oryzae [26]	Isopentyl	24	81.86	23.8	90.7
	Novozym 435 [32]	Isopentyl	3	~37	2.8	90.7
Homogeneous	Ammonium ferric sulphate[33]	Methyl	1.5	99.8	1.3	90.9
	Sulfuric acid [34]	Ethyl	1.7	~ 88	1.5	90.7
	Sulfuric acid [35]	Ethyl	2.9	~ 85	2.7	90.7
	Aryl imidazolium ionic liquid [36]	Propyl	4	93	3.8	92.1

Sulfuric acid[37]	Butyl	4	93	3.8	93.6

References

(1) Yanowitz, J.; Ratcliff, E. M. A.; Mccormick, R. L.; Taylor, J. D.; Murphy Battelle, M. J. Compendium of experimental cetane numbers; **2014**.

(2) Berg, P. S.; Pullen, M. D.; Barnard, D.; Vanderzyl, J. A liquid anti-pathogenic agricultural composition. WO 2020/144599 A1, **2020**.

(3) Berg, P. S.; Pullen, M. D.; Barnard, D.; Vanderzyl, J. An agricultural composition. WO 2020/144589 A2, **2020**.

(4) Han, Y.; Maria Smits, C. H.; Brennan, J.; Ian Page, G.; Teunis Pieter, J.; Dam, V. Animal feed additive and animal feed comprising alkyl esters of medium chain fatty acids, and their use in animal feed. US 2012/0029077 A1, **2012**.

(5) Farr, A.; Benz, K.; Kreutz, K.; Du¬-Val, D. Insect-repellent personal-care composition. WO 2023/076666 A1, **2023**.

(6) Douguet, M.; Picard, C.; Savary, G.; Merlaud, F.; Loubat-bouleuc, N.; Grisel, M. Spreading properties of cosmetic emollients: Use of synthetic skin surface to elucidate structural effect. *Colloids Surf. B Biointerfaces.* **2017**, *154*, 307–314.

(7) Gawas, S. D.; Jadhav, S. V.; Rathod, V. K. Solvent free lipase catalysed synthesis of ethyl laurate: optimization and kinetic studies. *Appl. Biochem. Biotechnol.* **2016**, *180 (7)*, 1428–1445.

(8) Welsh, F. W.; Murray, W. D.; Williams, R. E.; Katz, I. Microbiological and enzymatic production of flavor and fragrance chemicals. *Crit. Rev. Biotechnol.* **1989**, *9 (2)*, 105–169.

(9) Ledauphin, J.; Guichard, H.; Saint-Clair, J. F.; Picoche, B.; Barillier, D. Chemical and sensorial aroma characterization of freshly distilled calvados. 2. Identification of volatile compounds and key odorants. *J. Agric. Food Chem.* **2003**, *51 (2)*, 433–442.

(10) Guichard, H.; Lemesle, S.; Ledauphin, J.; Barillier, D.; Picoche, B. Chemical and sensorial aroma characterization of freshly distilled calvados. 1. Evaluation of quality and defects on the basis of key odorants by olfactometry and sensory analysis. *J. Agric. Food Chem.* **2003**, *51 (2)*, 424–432.

(11) Sousa, A.; Vareda, J.; Pereira, R.; Silva, C.; Câmara, J. S.; Perestrelo, R. Geographical differentiation of apple ciders based on volatile fingerprint. *Food Res. Int.* **2020**, *137*,109550.

(12) de Souza, P. P.; Cardeal, Z. de L.; Augusti, R.; Morrison, P.; Marriott, P. J. Determination of volatile compounds in brazilian distilled cachaça by using comprehensive two-dimensional gas chromatography and effects of production pathways. *J. Chromatogr. A.* **2009**, *1216 (14)*, 2881–2890.

(13) Api, A. M.; Belsito, D.; Botelho, D.; Bruze, M.; Burton, G. A.; Buschmann, J.; Cancellieri, M. A.; Dagli, M. L.; Date, M.; Dekant, W.; Deodhar, C.; Fryer, A. D.; Jones, L.; Joshi, K.; Kumar, M.; Lapczynski, A.; Lavelle, M.; Lee, I.; Liebler, D. C.; Moustakas, H.; Na, M.; Penning, T. M.; Ritacco, G.; Romine, J.; Sadekar, N.; Schultz, T. W.; Selechnik, D.; Siddiqi, F.; Sipes, I. G.; Sullivan, G.; Thakkar, Y.; Tokura, Y. RIFM fragrance ingredient safety assessment, ethyl laurate, CAS Registry Number 106-33-2. *FCT.* **2022**, *164*, 113099.

(14) Api, A. M.; Belsito, D.; Botelho, D.; Bruze, M.; Burton, G. A.; Cancellieri, M. A.; Chon, H.; Dagli, M. L.; Date, M.; Dekant, W.; Deodhar, C.; Fryer, A. D.; Jones, L.; Joshi, K.; Kumar, M.; Lapczynski, A.; Lavelle, M.; Lee, I.; Liebler, D. C.; Moustakas, H.; Na, M.; Penning, T. M.;

Ritacco, G.; Romine, J.; Sadekar, N.; Schultz, T. W.; Selechnik, D.; Siddiqi, F.; Sipes, I. G.; Sullivan, G.; Thakkar, Y.; Tokura, Y. RIFM fragrance ingredient safety assessment, ethyl decanoate, CAS Registry Number 110-38-3. *FCT*. **2022**. *167*, 113319.

(15) Lopes, A. C. A.; Andrade, R. P.; de Oliveira, L. C. C.; Lima, L. M. Z.; Santiago, W. D.; de Resende, M. L. V.; das Graças Cardoso, M.; Duarte, W. F. Production and characterization of a new distillate obtained from fermentation of wet processing coffee by-products. *J. Food Sci. Technol*. **2020**, *57 (12)*, 4481–4491.

(16) Anastopoulos, G.; Lois, E.; Zannikos, F.; Kalligeros, S.; Teas, C. HFRR Lubricity response of an additized aviation kerosene for use in CI engines. *Tribology International.* **2002**, *35(9),* 599-604.

(17) James-Meyer, L. S.; Coles, G. C. Lauric acid derivatives displaying inhibitory activity against gram-positive and/or gram-negative organisms lauric acid derivatives displaying inhibitory activity against gram-positive and/or gram-negative organisms. WO 2019/033125 Al, **2019**.

(18) Zoeller, M.; Opie, G. Cannabidiol formulation and methods of making and using, US 2020/0108014 Al, **2020**.

(19) Ley, K.; Orecchioni, M. Modulating immune response via targeting of olfactory receptor activity. WO 2019/204233 A1, **2019**.

(20) Talzi, V. P. A 13C and ^{1}H NMR Analysis of perfumes. *Russ. J. Appl. Chem.* 2006, *79 (1)*, 107–116.

(21) Myerson, J.; Haddonl, W. F.; Soderstrom, E. L. Sec-butyl (Z)-7-tetradecenoate. A novel sex pheromone component from the western grapeleaf skeletonizer, harrsina brillians, *TETL*. **1982**, *23*, 2757-2760.

(22) Dey, P.; Ray, S.; Chaudhuri, T. K. Immunomodulatory activities and phytochemical characterisation of the methanolic extract of dioscorea alata aerial tuber. *J. Funct. Foods*. **2016**, *23*, 315–328.

(23) Pasinato, R. Lubricating product for circular textile machinery for hosiery. WO 2015/159314 Al, **2015**.

(24) Vassilev, D.; Petkova, N.; Koleva, M.; Denev, P. Ultrasound-assisted method for the synthesis of tertiary fatty aliphatic esters with potential antimicrobial activity. *Biointerface Res. Appl. Chem.* **2020**, *10 (6)*, 6829–6836.

(25) Blank, I. K. O.; Blank, A. H. Pharmaceutical compositions for topical application. EP0712633A1, **1996**.

(26) Vilas Bôas, R. N.; Ceron, A. A.; Bento, H. B. S.; de Castro, H. F. Application of an immobilized *Rhizopus oryzae* lipase to batch and continuous ester synthesis with a mixture of a lauric acid and fusel Oil. *Biomass and Bioenergy*. **2018**, *119*, 61–68.

(27) Gallego-Villada, L. A.; Alarcón, E. A.; Palermo, V.; Vázquez, P. G.; Romanelli, G. P. Kinetics for the biodiesel production from lauric acid over Keggin heteropolyacid loaded in silica framework. *JIEC*. **2020**, *92*, 109–119.

(28) Zatta, L.; Ramos, L. P.; Wypych, F. Acid-activated montmorillonites as heterogeneous catalysts for the esterification of lauric acid acid with methanol. *Appl. Clay Sci.* **2013**, *80–81*, 236–244.

(29) Wawrzyńczak, A.; Jarmolińska, S.; Nowak, I. Nanostructured KIT-6 materials functionalized with sulfonic groups for catalytic purposes. *Catal. Today.* **2022**, *397–399*, 526–539.

(30) Solarte, C.; Yara-Varón, E.; Eras, J.; Torres, M.; Balcells, M.; Canela-Garayoa, R. Lipase activity and enantioselectivity of whole cells from a wild-type *Aspergillius flavus* strain. *J. Mol. Catal. B Enzym.* **2014**, *100*, 78–83.

(31) Vassiliadi, E.; Xenakis, A.; Zoumpanioti, M. Chitosan Hydrogels: A new and simple matrix for lipase catalysed biosyntheses. *Mol. Catal.* **2018**, *445*, 206–212.

(32) Varma, M. N.; Madras, G. Synthesis of isoamyl laurate and isoamyl stearate in supercritical carbon dioxide. *Appl. Biochem. Biotechnol.* **2007**, *141*, 139–147.

(33) Ganesan, S.; Nadarajah, S.; Khairuddean, M.; Teh, G. B. Studies on lauric acid conversion to methyl ester via catalytic esterification using ammonium ferric sulphate. *Renew Energy*. **2019**, *140*, 9–16.

(34) Murad, P. C.; Hamerski, F.; Corazza, M. L.; Luz, L. F. L.; Voll, F. A. P. Acid-catalyzed esterification of free fatty acids with ethanol: an assessment of acid oil pretreatment, kinetic modeling and simulation. *Reac. Kinet. Mech. Cat.* **2018**, *123 (2)*, 505–515.

(35) Margarida, B. R.; Flores, L. I.; Hamerski, F.; Voll, F. A. P.; Luiz, L. F. , Simulation, optimization, and economic analysis of process to obtain esters from fatty acids. *Biofuels, Biofpr*. **2021**, *15 (3)*, 749–769.

(36) Thul, M.; Pantawane, A.; Lin, W.; Lin, Y. J.; Su, P. F.; Tseng, S. A.; Wu, H. R.; Ho, W. Y.; Luo, S. Y. Tunable aryl imidazolium ionic liquids (TAILs) as environmentally benign catalysts for the esterification of fatty acids to biodiesel fuel. *Catal. Commun*. **2021**, *149*, 106243.

(37) Juan, J. C.; Zhang, J.; Yarmo, M. A. 12-Tungstophosphoric acid supported on MCM-41 for esterification of fatty acid under solvent-free condition. *J. Mol. Catal. A Chem*. **2007**, *267 (1–2)*, 265–271.

(38) American Oil Chemists' Society. American Oil Chemists' Society (AOCS). https://www.aocs.org/attain-lab-services/methods/methods/search-results?method=111545 (accessed 2025-02-18).

(39) Skiera, C.; Steliopoulos, P.; Kuballa, T.; Diehl, B.; Holzgrabe, U. determination of free fatty acids in pharmaceutical lipids by ^{1}H NMR and comparison with the classical acid value. *J. Pharm. Biomed. Anal.* **2014**, *93*, 43–50.

(40) Satyarthi, J. K.; Srinivas, D.; Ratnasamy, P. Estimation of Free Fatty Acid Content in Oils, Fats, and Biodiesel by ^{1}H NMR Spectroscopy. *Energy and Fuels.* **2009**, *23 (4)*, 2273–2277.

(41) Marchetti, J. M.; Errazu, A. F. Comparison of different heterogeneous catalysts and different alcohols for the esterification reaction of oleic acid. *Fuel*. **2008**, *87 (15–16)*, 3477–3480.

(42) McMurry John. *Organic Chemistry*, 7th ed.; Cengage Learning: USA, **2008**.
